# Effectiveness of Haemodiafiltration with Heat Sterilized High-Flux Polyphenylene HF Dialyzer in Reducing Free Light Chains in Patients with Myeloma Cast Nephropathy

**DOI:** 10.1371/journal.pone.0140463

**Published:** 2015-10-14

**Authors:** Mathieu Rousseau-Gagnon, Mohsen Agharazii, Sacha A. De Serres, Simon Desmeules

**Affiliations:** 1 Division of Nephrology, Department of Medicine, CHU de Quebec-Hôtel-Dieu de Québec, Quebec, Canada; 2 Department of Medicine, Faculty of Medicine, Université Laval, Québec, Québec, Canada; University of Sao Paulo Medical School, BRAZIL

## Abstract

**Introduction:**

In cases of myeloma cast nephropathy in need of haemodialysis (HD), reduction of free light chains using HD with High-Cut-Off filters (HCO-HD), in combination with chemotherapy, may be associated with better renal recovery. The aim of the present study is to evaluate the effectiveness of haemodiafiltration (HDF) in reducing free light chain levels using a less expensive heat sterilized high-flux polyphenylene HF dialyzer (HF-HDF).

**Methods:**

In a single-centre prospective cohort study, 327 dialysis sessions were performed using a 2.2 m^2^ heat sterilized high-flux polyphenylene HF dialyzer (Phylther HF22SD), a small (1.1m^2^) or large (2.1 m^2^) high-cut-off (HCO) dialyzer (HCO_S_ and HCO_L_) in a cohort of 16 patients presenting with dialysis-dependent acute cast nephropathy and elevated free light chains (10 kappa, 6 lambda). The outcomes of the study were the mean reduction ratio (RR) of kappa and lambda, the proportion of treatments with an RR of at least 0.65, albumin loss and the description of patient outcomes. Statistical analysis was performed using linear and logistic regression through generalized estimating equation analysis so as to take into account repeated observation within subjects and adjust for session duration.

**Results:**

There were no significant differences in the estimated marginal mean of kappa RR, which were respectively 0.67, 0.69 and 0.70 with HCO_L_-HD, HCO_S_-HDF and HF-HDF (P = 0.950). The estimated marginal mean of the proportions of treatments with a kappa RR ≥0.65 were 68%, 63% and 71% with HCO_L_-HD, HCO_S_-HDF and HF-HDF, respectively (P = 0.913). The estimated marginal mean of lambda RR were higher with HCO_L_-HDF (0.78), compared to HCO_L_-HD and HF-HDF (0.62, and 0.61 respectively). The estimated marginal mean proportion of treatments with a lambda RR ≥0.65 were higher with HCO_L_-HDF (81%), compared to 57% in HF-HDF (P = 0.042). The median albumin loss were 7, 21 and 63 g/session with HF-HDF, HCO_L_-HD and HCO_L_-HDF respectively (P = 0.044). Among survivors, 9 out of 10 episodes of acute kidney injuries became dialysis-independent following a median time of renal replacement therapy of 40 days (range 7–181).

**Conclusion:**

Therefore, in patients with acute dialysis-dependent myeloma cast nephropathy, in addition to chemotherapy, HDF with a heat sterilized high-flux polyphenylene HF dialyzer could offer an alternative to HCO dialysis for extracorporeal kappa reduction with lower albumin loss.

## Introduction

Multiple myeloma (MM) is a malignant monoclonal proliferation of plasma cells that usually produces an excess of free light chain (FLC). Myeloma cast nephropathy (CN), the most frequent cause of dialysis-dependent acute kidney injury (AKI), occurs when filtered FLCs precipitate with uromodulin, causing obstruction of the tubules [1]. Among patients with CN, requirement for chronic renal replacement therapy (RRT) is associated with poor clinical outcomes [2]. The single major randomized controlled trial of plasma exchange did not show significant clinical benefits and FLC levels were not measured[3]. More recently, Brunette et al. have reported an 86% renal recovery rate in 14 patients treated with bortezomib and plasma exchange[4]. However, removal of FLC by plasmapheresis has usually been considered inefficient due to the relatively limited volume of plasma as compared to the FLC’s high volume of distribution[3, 5].

Conventional haemodialysis (HD) fails to reduce FLC levels significantly given their high molecular weight (monomeric kappa of 22.5 kDa, dimeric lambda of 45 kDa) [6]. Recently, haemodialysis with High-Cut Off dialyzers (HCO-HD), which are highly permeable to low molecular weight proteins of up to 50 kDa, have been shown to achieve a significant reduction in post-dialysis serum FLC levels. Indeed, haemodialysis with a HCO-1100 dialyzer, which has a membrane surface area of 1.1 m^2^, was effective in reducing both kappa and lambda FLC levels. However, by using 2 HCO-1100 dialyzers in series, and therefore doubling the membrane surface area, there was a greater increase in FLC clearance and FLC reduction ratios [7]. Subsequently, it was shown that patients who received effective chemotherapy regimens and adjunctive HCO-HD had a much better renal recovery rate than historical controls [8–10]. This improvement was achieved by using 2 HCO-1100 dialyzers in series for each dialysis session, and required albumin infusion to compensate for the increased loss of albumin. Consequently, HCO-1100 dialyzers were discontinued by Gambro and were replaced by Theralite, an HCO dialyzer with a membrane surface area of 2.1 m^2^. Randomized-control trials are ongoing, but even if results are positive, the high cost of HCO filter are likely to limit widespread availability of HCO-HD [11, 12].

Enhancing convective clearance through haemodiafiltration (HDF) has been shown to be more efficient than conventional HD to reduce the levels of middle-size molecules [13]. Indeed, in a recent study where high-efficiency HDF was associated with reduced all-cause mortality compared to conventional haemodialysis, HDF resulted in kappa FLC reduction ratios, which were comparable to those achieved with HCO-HD [14]. Nevertheless, the effectiveness of HDF to improve survival in the general end-stage kidney disease patients still remains controversial [13–17].

Therefore, in a cohort of patients with dialysis-dependant myeloma cast nephropathy and elevated levels of FLC, we sought to determine the comparative effectiveness of HDF in reducing kappa and lambda FLC levels using a heat sterilized high-flux polyphenylene HF dialyzer (HF-HDF), and HCO-HD and HCO-HDF. We also determined the extent of albumin loss and describe the patient outcomes of this cohort.

## Materials and Methods

### Study design and population

This is a single-center longitudinal prospective observational study. Between May 2009 and July 2012, all subjects who started dialysis following a working diagnosis of AKI due to CN were eligible. CN was confirmed by renal biopsy, unless declined or contraindicated due to concurrent antiplatelet therapy (5 patients). The diagnosis of AKI was based on a combination of rising creatinine, non-atrophic kidneys as evaluated by ultrasound, histological examination or subsequent renal recovery. Indication for initiation of dialysis were an eGFR of <10 mL/min/1.73m^2^ with associated clinical symptoms. Exclusion criteria for extracorporeal reduction of FLC were AKI with elevated FLC not needing replacement therapy, absence of CN on the renal biopsy, and plasma exchange. Serum FLC levels were prospectively measured before and after each session to determine FLC reduction ratio (RR). Clinical data were prospectively collected and no patient was lost to follow-up. The chemotherapy regimen was recorded. The project was evaluated and approved by the *Comité d’Éthique de la Recherche du CHU de Québec* as a dialysis quality assurance evaluation. All data were anonymized as requested by the ethics committee.

### Choice of dialyzer

In a preliminary study in patients with myeloma cast nephropathy, we observed a kappa reduction ratio (RR) with various synthetic high flux membranes during HDF were: 0.16 with 2.1 m^2^ polyarylethersulfone/ polyvinylpyrrolidone/polyamide (Polyflux 210H) (n = 36), 0.27 with 2.5 m^2^ polysulfone (F250) (n = 2), 0.45 with 2.1 m^2^ AN-69 ST (Nephral-500) (n = 8) and 0.62 with 2.2 m^2^ heat sterilized high-flux polyphenylene HF (Phylther HF22SD) (n = 5). For the lambda FLC, HDF with heat sterilized high-flux polyphenylene HF (Phylther HF22SD) achieved a mean RR of 0.35 (n = 3), while an HDF with a 2.1 m^2^ HCO (Theralite) achieved a mean RR of 0.54 (n = 12).

Therefore, for kappa FLC, we preferentially performed HDF with heat sterilized high-flux polyphenylene HF (HF22SD) over HCO dialyzers (small 1.1 m^2^ HCO-1100 (HCO_S_) or large 2.1m^2^ Theralite (HCO_L_)). For patients with lambda FLC, because the preliminary analysis suggested a lower lambda RR with HDF with heat sterilized high-flux polyphenylene HF (Phylther HF22SD), we performed a maximum of 35 HDF treatments with large HCO dialyzer. After the initial 35 sessions, because of budgetary constraints, all patients were switched to HDF with the heat sterilized high-flux polyphenylene HF (Phylther HF22SD). In the case of inability to perform HDF, HD was performed with HCO dialyzers. The characteristics and performances of the two dialyzers included in this study are shown in Table 1 [18].

**Table 1:** Dialyzers characteristics and performance.

Dialyzer	Phylther* HF22SD	Theralite
**Material**	Polyphenylene HF	PAES/PVP
**Area (m** ^**2**^ **)**	2.2	2.1
**Flux**	High-Flux (HF)	High Cut-Off (HCO)
**Molecular weight cut-off (kDa)**	34–36	170–320
**Molecular weight retention onset (kDa)**	12–14	15–20
**Pore size (nm)**	6–8	8–12
**Sieving Coefficient**		
** β-2 microglobuline (11.8 kDa)**	0.93	0.95
** Albumin (66.5 kDa)**	0.003	0.2

*Heat sterilized

PAES: polyarylethersulfone; PVP: polyvinylpyrrolidone.

### Modalities of renal replacement therapy

From 2009 to March 2011, online HDF was performed with a single Gambro’s AK 200 Ultra S dialysis system (Gambro, Lund, Sweden). When water quality standards were not met or machine was unavailable, convective therapy was performed with Gambro’s Integra using Hemosol-BO 5-liter bags as sterile infusion solution. Since March 2011, all online HDF treatments are done with Gambro’s Artis dialysis system.

Renal replacement therapy was performed daily or alternate daily using a single dialyzer. Session length ranged from 210 to 360 minutes. Blood flow (Qb) was between 300 and 400 mL/min and dialysate flow was 700–800 mL/min. As mentioned above and based on clinical availability, HDF was done with 3 different Gambro systems: 1) AK 200 Ultra S performed pre-dilution online HDF with an infusion flow between 125 and 175 mL/min, 2) Integra system allowed post filter infusion of sterile Hemosol-BO at a rate of 50 mL/min and 3) the Artis system maximized post filter infusion using pressure control mode except with HCO dialyzers, where volume controlled post-dilution with an infusion between 70 and 90 mL/min was used. Infusion volumes were recorded at the end of each session. Low volume HDF was defined as an infusion volume per session of less than 15 L while more than 15 L of post filter infusion was qualified as high volume [19]. When heparin anticoagulation was contraindicated, saline flushes or regional citrate anticoagulation were performed. Albumin repletion was performed on clinical basis. Renal replacement was performed until renal recovery as defined below.

### Outcome measures

The primary outcomes of the study were mean FLC reduction ratios and percentages of treatments with a RR≥0.65 [8]. We examined albumin loss as secondary outcome, and we described patient survival and renal recovery. Recovery of renal function was defined as renal function sufficient to stop dialysis. Dialysis was continued until an estimated glomerular filtration rate (MDRD eGFR) of > 10 mL/min was achieved in combination with a pre-dialysis FLC level of <500–1000 mg/L, depending on the initial levels of FLC at presentation of AKI.

### Biological parameters

Serum FLC, as well as β2-microglobulin and creatinine were measured before and after HDF/HD sessions using the slow-flow/stop-pump technique. Serum FLC was measured by nephelometry using a particle-enhanced, highly specific, homogeneous immunoassay (FREELITE, The Binding Site, San Diego, USA). β2-microglobulin was measured using nephelometry using a specific reactive (BN Prospect, Siemens AG, Munich, Germany). Albumin loss was estimated with a continuous partial collection (20 mL/h) of spent dialysate. Albumin levels in spent dialysate were assessed with a turbidometric immunoassay (Beckman-Coulter DCX600, Mississauga, Canada).

### Statistical analysis

Values are reported as mean (SD) or median (25–75th percentiles) unless specified otherwise. To obtain an estimate of the impact of the dialysis-filter modality on the RR of FLC, a generalized estimating equation (GEE) analysis was used to take into account repeated measures within each individual. To estimate the effect of HDF modality (predilution, low-efficiency post-dilution and high-efficiency post-dilution) on the FLC RR, we used a GEE model to report the estimated marginal means adjusted for the duration of dialysis session. To estimate time adjusted marginal means of the proportion of patients who achieved a FLC RR of ≥0.65, a GEE model was used to take into account repeated measures within each patients. To estimate time adjusted marginal means of the proportion of patients who achieved a FLC RR of ≥0.65, a GEE model was used to take into account repeated measures within each patients. In the examination of the proportion of treatments with lambda RR ≥0.65, it was not possible to estimate the proportions for the HCO_L_-HD group as all 4 treatments achieved an RR≥0.65 and therefore the convergence criteria were not satisfied. Therefore, the estimates of the proportions of lambda RR ≥0.65 were obtained for HCO_L_-HDF and HF-HDF by excluding the HCO_L_-HD group for this analysis. Bonferroni correction method was used to adjust for multiple comparisons. The S1 Dataset that was used for the analysis is available in the Supporting Information files. Data were analysed using SPSS 22. A two-tailed P-value of <0.05 was considered statistically significant.

## Results

### Clinical characteristics

Extracorporeal FLC reduction was studied in 16 patients who had 17 episodes of AKI requiring RRT due to probable or confirmed CN. Overall, 8 episodes were due to relapsing MM after at least one course of chemotherapy. At the presentation of AKI, the median age was 67 years and the median initial serum creatinine level was 692 μmol/L (range: 383–1400). Description of the clinical baseline and individual characteristics and outcomes are presented in Table 2. Kappa and lambda monoclonal FLC accounted for 10 and 6 patients respectively.

**Table 2:** Clinical and biochemical characteristics.

	n = 16
**Age (y)**	67 (46–79)
**AKI Episodes (n)**	17*
**Baseline serum creatinine (μmol/L)**	85 (50–195)
**Serum creatinine at presentation (μmol/L)**	692 (383–1400)
**Kappa MM (n)**	10
** kappa FLC (mg/L)**	6 050 (825–35 500)
**Lambda MM (n)**	6
** lambda FLC (mg/L)**	11 600 (2 620–14 100)
**Total analyzed RRT sessions**	324
**Number of RRT sessions/subject**	18 ± 12
**Number of 25% albumin units used**	39
**Baseline serum albumin (g/L)**	29±6.8
**Minimum serum albumin (g/L)**	21±6.3
**Clinical outcome**	
** Renal recovery- n (%)**	9 (53%)
** Time to Renal Recovery (d)**	40 (7–181)
** Dialysis dependant**	1 (6%)
** Death–n (%)**	7 (41%)
** Time to Death (d)**	36 (6–84)
**Follow-up Renal Function at 6 Months**	
** Creat (μmol/L)**	267 (53–507)
** Range of eGFR (mL/min/1.73m** ^**2**^ **)**	10–90
**Biopsy**	
** CN**	9
** CN+Amyloidosis**	1
** CN+LCDD**	1
** Biopsy declined or contraindicated**	5
**Chemotherapy** *	
** Bortezomib-Dexamethasone**	12
** Revlimid-Dexamethasone**	5
** Refused chemotherapy**	1

CN: Myeloma Cast Nephropathy;LCDD: Light Chain Deposition Disease; MM: Multiple Myeloma; AKI: acute kidney injury. Values are mean ±SD or median (range)

* 1 subject had two distinct episodes of AKI

One patient received bortezomid- and Revlimid-based regimen. Another patient with two episodes of AKI received Bortezomib and Revlimid respectively for the first and second episode.

### Reduction ratio of kappa FLC

Overall, 203 RRT sessions were analysed to determine kappa RR. Table 3 shows the mean kappa RR per patient and treatment protocol.

**Table 3:** Kappa free light chain reduction ratio per patient and treatment protocol.

Patient	RRT Modality	kappa RR	Number of RRT
**k-1**	HF-HDF	0.82±0.08	26
**k-2**	HCO_L_-HD	0.69±0.08	25
**k-3**	HF-HDF	0.37±0.10	13
**k-4**	HF-HDF	0.78±0.06	17
**k-5**	HF-HDF	0.67±0.07	8
**k-6**	HCO_S_-HDF	0.71±0.06	13
	HF-HDF	0.73±0.02	3
**k-7**	HF-HDF	0.47±0.11	26
**k-8**	HF-HDF	0.78±0.22	10
**k-9a**	HF-HDF	0.86±0.02	6
**k-9b**	HF-HDF	0.90±0.03	20
**k-10**	HF-HDF	0.62±0.12	36

Values are mean±SD

RRT: Renal replacement therapy;HF-HDF: Haemodiafiltration with heat sterilized Polyphenylene HF of 2.2 m^2^; HCOs-HDF: Haemodiafiltration with high cut-off small surface area membrane (1.1 m^2^); HCO_L_-HD: Hemodialysis with high cut-off large surface area membrane (2.1 m^2^).

**Fig 1:**
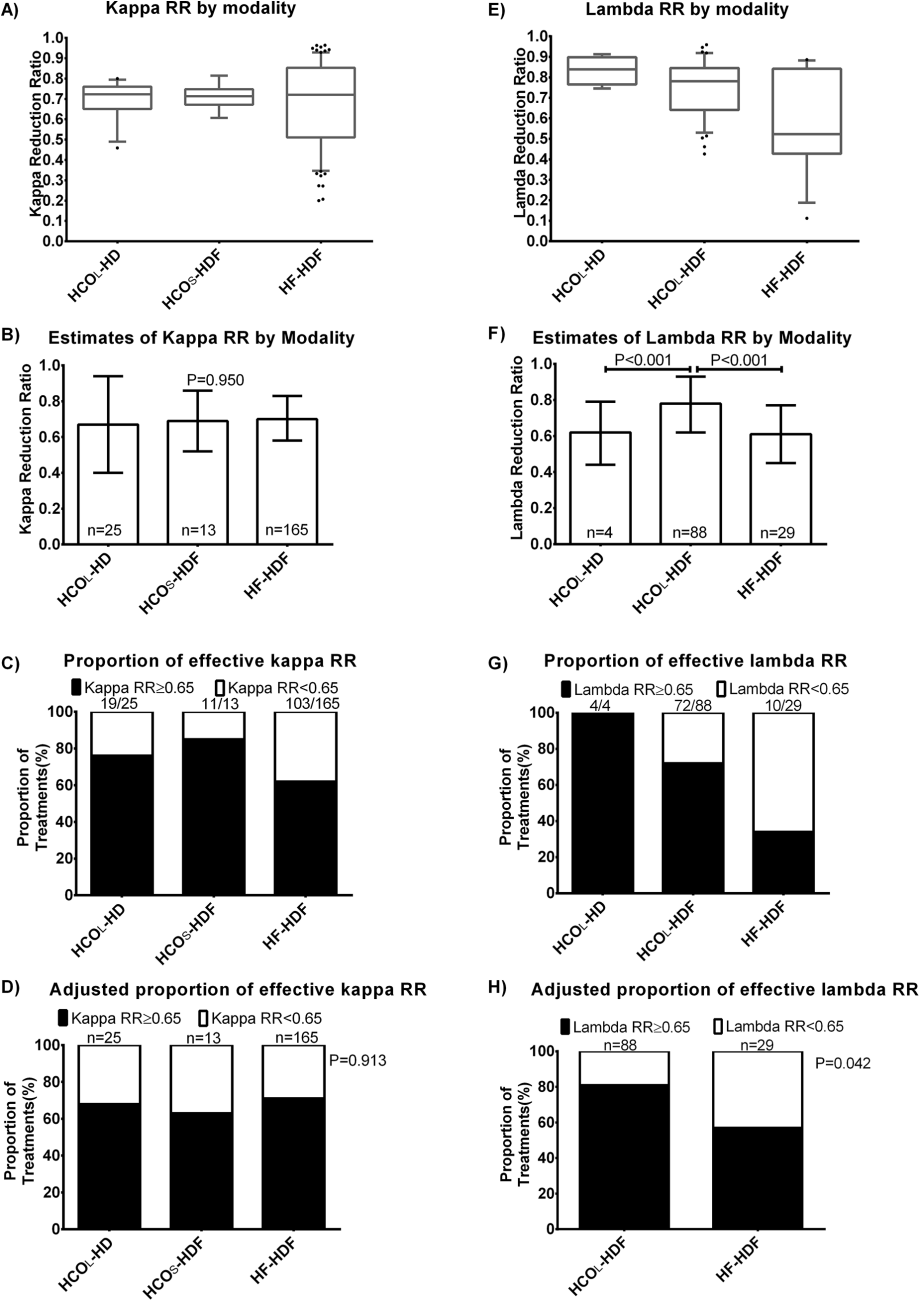
Free light chain reduction ratio by modality of renal replacement therapy. The left panel shows A) the crude reduction ratio (RR) of kappa free light chain by haemodialysis (HD) or haemodiafiltration (HDF) using large (2.1 m^2^) high cut-off filters (HCO_L_), small (1.1 m^2^) high cut-off filters (HCO_S_), and the 2.2 m^2^ heat sterilized high-flux polyphenylene HF (HF). Panel B shows the estimated marginal means of kappa reduction ratio taking into account repeated measurements within individuals and session duration using a GEE model. The percentages of the treatments with a kappa reduction ratio of ≥ 0.65 with each modality is shown in panel C. Panel D shows the percentages of the treatments with a kappa reduction ratio of ≥ 0.65 with each modality taking into account repeated measurements within individuals and session duration using a GEE model. The right panel shows the crude RR of lambda free light chain by renal replacement modality (E), and the estimated marginal means of lambda RR (F) taking into account repeated measures within individuals and session duration using GEE model. The crude and adjusted (within subjects and session duration) percentage of treatments with a lambda RR ≥0.65 are respectively shown in panels G and H. All p-values take into account adjustments for multiple comparisons using Bonferroni method.

**Fig 2:**
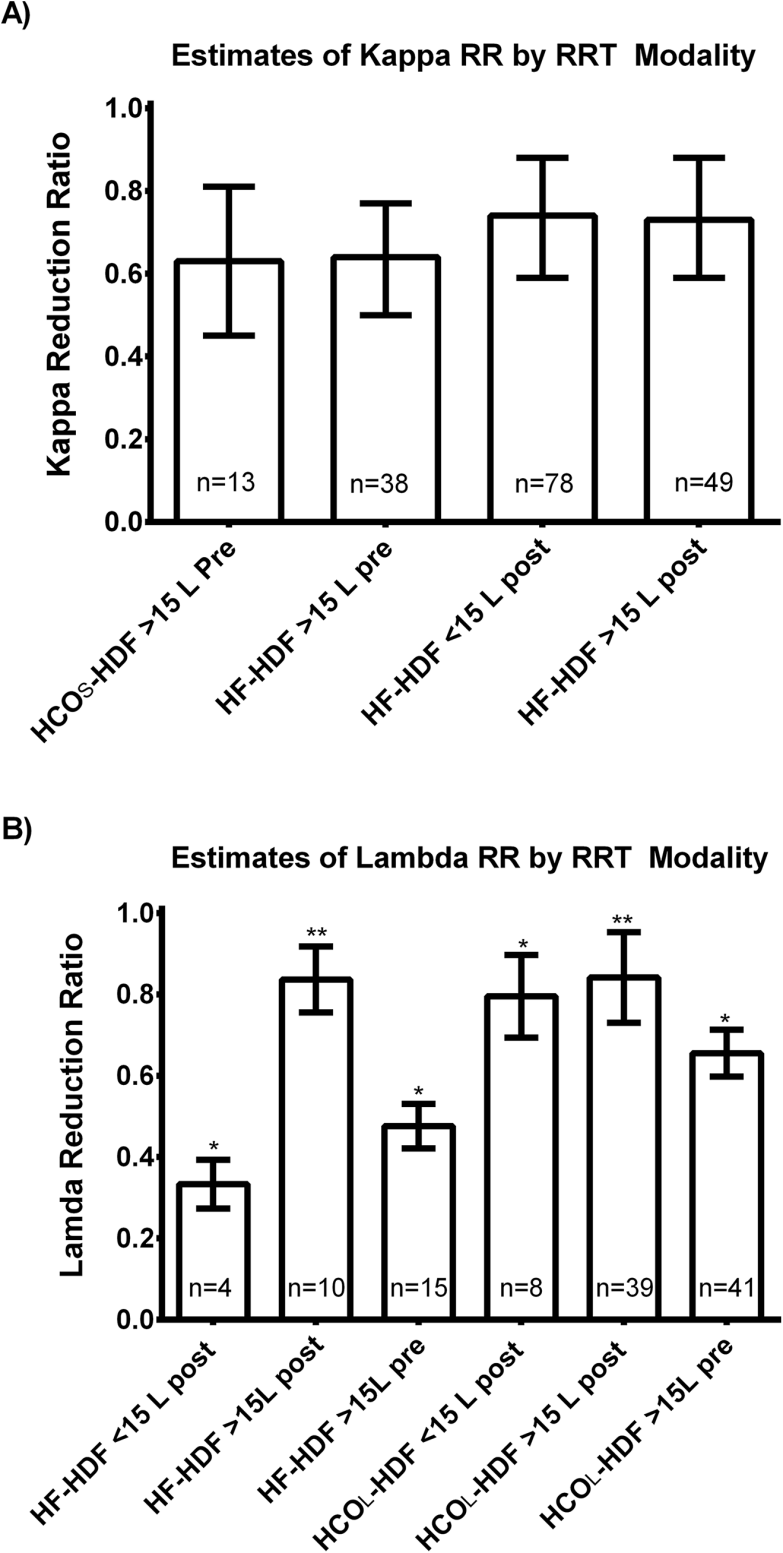
Impact of haemodiafiltration method of free light chain reduction ratio. The impact of haemodiafiltration (HDF) in predilution (Pre), low-efficiency post-dilution (<15L post), and high-efficiency post-dilution (>15L post) are performed using large (2.1 m^2^) high cut-off filters (HCO_L_), small (1.1 m^2^) high cut-off filters (HCO_S_), and the 2.2 m^2^ heat sterilized high-flux polyphenylene HF (HF). The estimated marginal means of kappa (A) and lambda (B) light chain reduction ratio taking into account repeated measures and duration of each treatment session. P-values reported are adjusted for multiple comparisons using the Bonferroni correction method.* indicates that values are statistically different from other groups (P<0.01), ** indicates that values are statistically not different compared to other groups.

Fig 1A shows a box-plot graph of the kappa RR according to the RRT modality. Fig 1B shows that the estimates of the kappa RR were similar among the three different modalities taking into account repeated measurements within patients and duration of the session. There were no significant differences in the estimated marginal mean of kappa RR, which were 0.67, 0.69 and 0.70 with HCO_L_-HD, HCO_S_-HDF and HF-HDF respectively (P = 0.950). Fig 1C shows the crude percentage of treatment episodes where a kappa RR of more than 0.65 was achieved. Fig 1D shows the adjusted percentages of treatment episodes where a kappa RR of ≥0.65 was achieved, taking into account repeated measurements and duration of the session. The estimated marginal mean of the proportions of treatments with a kappa RR ≥0.65 were 68%, 63% and 71% with HCO_L_-HD, HCO_S_-HDF and HF-HDF respectively (P = 0.913). Fig 2A shows that the modality of haemodiafiltration and the volume did not significantly affect the kappa RR in this group of patients.

### Reduction ratio of lambda FLC

In total, 121 RRT sessions were analysed for lambda FLC RR. Table 4 shows the mean lambda RR per patient and treatment protocol.

**Table 4:** Lambda free light chain reduction ratio per patient and treatment protocol.

Patient	RRT Modality	Lambda RR	Number of RRT
**l-1**	HCO_L_-HDF	0.84±0.09	20
**l-2**	HF-HDF	0.31±0.15	4
	HCO_L_-HDF	0.69±0.09	6
**l-3**	HF-HDF	0.85±0.03	10
	HCO_L_-HD	0.83±0.07	4
	HCO_L_-HDF	0.91±0.05	5
**l-4**	HF-HDF	0.47±0.10	15
	HCO_L_-HDF	0.65±0.11	35
**l-5**	HCO_L_-HDF	0.81±0.02	3
**PD**	HCO_L_-HDF	0.79±0.04	19

Values are mean±SD

RRT: Renal replacement therapy;HF-HDF: Haemodiafiltration with heat sterilized Polyphenylene HF of 2.2 m^2^; HCO_L_-HDF: Haemodiafiltration with high cut-off large surface area membrane (2.1 m^2^); HCO_L_-HD: Hemodialysis with high cut-off large surface area membrane (2.1 m^2^).

Fig 1E shows a box-plot graph of the lambda RR according to the RRT modality. Fig 1F shows that the estimates of the lambda RR were similar between HF-HDF and HCO_L_-HD, when taking into account repeated measures within each individual and duration of each session. The estimated marginal mean of lambda RR were higher with HCO_L_-HDF (0.78) as compared to HCO_L_-HD and HF-HDF (0.62, and 0.61 respectively). Fig 1G shows the crude percentage of treatment episodes where a lambda RR of more than 0.65 was achieved. Fig 1H shows that a higher proportion of treatments achieved a lambda RR ≥0.65 with HCO_L-_HDF as compared to HF-HDF, after adjusting for repeated measures and duration of each session. The estimated marginal mean proportion of treatments with a lambda RR ≥0.65 were higher with HCO_L_-HDF (81%) as compared to 57% in HF-HDF (P = 0.042). Fig 2B shows that high-efficiency post-dilution provided the highest lambda RR.

**Fig 3:**
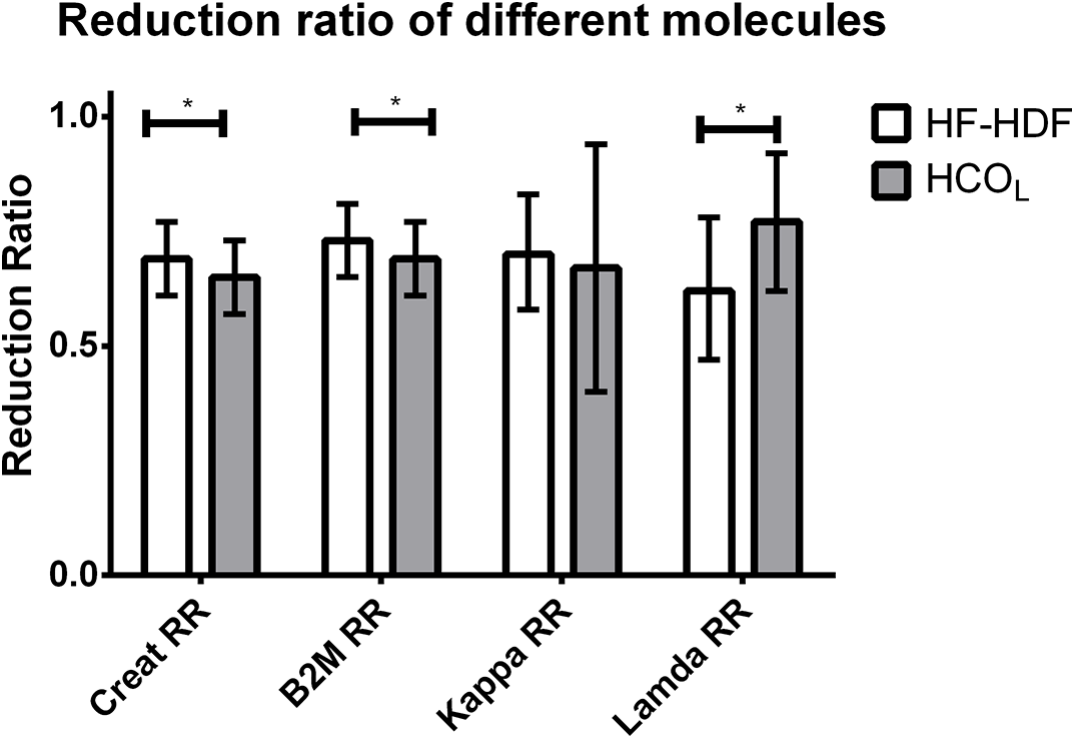
Reduction ratio of molecules of increasing molecular weights. The figure shows the reduction ratio (RR) of creatinine (113 Da), β-2 microglobulin (β2M, 11.8kDa), kappa (22.5 kDa) and lambda (45 kDa) free light chains, using haemodiafiltration with heat sterilized high-flux polyphenylene HF (HF-HDF), compared to haemodialysis or haemodiafiltration with a large (2.1 m^2^) high-cut-off dialyzer (HCO_L_). Estimates are obtained by generalized estimating equation taking into account repeated measures and session duration. * indicates a P-value of <0.05.

Taking into account repeated measures and session duration by using generalized estimating equation, Fig 3 shows the reduction ratio of creatinine, β-2 microglobuline, kappa and lambda free light chains, using HF-HDF compared to the pooled data from HCO_L_-HD and HCO_L_-HDF.

### Albumin loss

Albumin losses were 7 (6–9.6), 21 (12–23), and 63 (48–66) g/session with HF-HDF, HCO_L_-HD and HCO_L_-HDF respectively (P = 0.044). Overall, 6 subjects received a total of 39 units of albumin. Patients who received more than 70% of their treatments with an HCO filter received 27 units of albumin.

### Renal recovery

During the follow-up, in the 7 patients who died, the causes of death were infection (n = 2), progressive MM (n = 3), treatment refusal (n = 1) and bilateral subdural hematoma (n = 1), with a median time to death of 36 days (range: 6–84 days). Renal function improved sufficiently to wean dialysis in all but one survivor. This patient, in whom both amyloidosis and CN were present on the kidney biopsy remained dialysis-dependent. One patient was treated for two episodes of CN and recovered on both occasions after 7 and 34 days of RRT. Median length of RRT among survivors was 40 d (range 7–181). Two patients were transferred to another facility for follow-up. At 6 months, all surviving patients remained dialysis-free with a median creatinine of 267 μmol/L (range: 53–507).

## Discussion

The results of our study suggest that kappa FLC can be reduced effectively with a heat sterilized high-flux polyphenylene HF using haemodiafiltration. Using HF-HDF, kappa FLC RRs with the 2.2 m^2^ heat sterilized high-flux polyphenylene HF (Phylther HF22SD) compares favourably with the results published in the landmark study of Hutchison and colleagues [8], which showed a median RR for kappa light chains of 0.69 using dual (2 x 1.1 m^2^) HCO-1100 dialyzers in series for an 8-hour dialysis session. In agreement with the findings from the Hutchison’s study [8], the majority of survivors from our cohort recovered sufficient renal function to be weaned off dialysis.

Our results are in keeping with a recent study showing better FLC RR with HDF when compared to HD [20]. Sieving coefficients of high-flux dialyzers for molecules up to 15 kDa are somewhat similar, suggesting that the ability of the heat sterilized polyphenylene HF to reduce kappa FLC could be due to either adsorptive property specific to the membrane, or a rapid decline in pore size for molecular weights between 15 to 35 kDa. Although the contribution of FLC adsorption to the reduction ratio of kappa FLC with the polyphenylene HF dialyzer was not tested directly in this study, the marked difference in kappa and lambda (22.5 kDa *vs* 45 kDa) reduction ratios suggest a major role of the pore size hypothesis. However, our results cannot be generalized to other dialyzers made with the same material, as the sterilisation process can affect membrane characteristics [21]. Therefore, heat-sterilised polyphenylene HF may be more porous than gamma sterilised polyphenylene HF, and this distinction should be further studied before further generalization. We also observed good lambda chain removal with heat-sterilised polyphenylene HF using high-volume post-dilution HDF.

Nevertheless, because of the heterogeneity in kappa and lambda RR ratio between individuals, it is possible that the existence of free light chains, either as monomers or dimers, affect the molecular weight and therefore affect the sieving coefficient. Indeed, since lambda free light chains are generally present as dimers (45 kDa), we did not expect high-flux dialyzers to clear proteins of this size effectively. Excellent RR of lambda free light chain by HF-HDF in some patients may suggest that lambda free light chain might have been present as monomers. On the other hand, lower kappa FLC RR by HF-HDF may suggest multimerization of the kappa FLC, as described in a recent publication [22]. Thus, irrespective of the method used, monitoring the efficiency of free light chain RR is essential as both types of free light chains can be present as multimers.

The addition of HDF to large HCO dialyzers numerically improved lambda RR. However, this improvement in lambda RR was associated with a clinically significant three-fold increase in albumin loss (63g/session) [23]. As the proposed benefit of FLC lowering should initially be maintained by daily dialysis, this level of albumin loss seems prohibitive.

While a formal pharmacoeconomic analysis is beyond the scope of the present study, our findings suggest that HDF with heat sterilized polyphenylene HF could be cost effective to reduce kappa FLC, considering the very high cost of the HCO dialyzers (CAD $800 vs $9). Solely based on dialyzers costs, using HF-HDF instead of HCO-HD among patients with kappa FLC elevation and a median of 17 sessions before renal recovery could translate into savings of more than CAD$10 000 per patient. Furthermore, systematic albumin replacement is not mandatory, as albumin loss with HF-HDF is at least threefold lower than HCO dialyzers. However, more recently, the use of supra-hemodiafiltration with endogenous reinfusion of the regenerated ultrafiltrate by a sorbent cartridge, has been shown to reduce kappa and lambda free light chains without the need for albumin replacement in 4 patients [24].

Our cohort included a limited number of patients, which limits the conclusion for some modalities. As the renal replacement modality was limited by the availability of filters and haemodiafiltration, individual patients (FLC clones) were not exposed to every treatment modality. However, because of the nature of the outcome measured, the observational design with serial observations on a group of patients offered the opportunity to test the hypothesis adequately using models adjusted for repeated measures. Uromodulin affinity of FLC is variable [25] and our decision to pursue RRT until pre dialysis FLC were less than 500–1000 mg/L might have resulted in unnecessary RRT sessions in some patients. Time to renal recovery may therefore be longer in our cohort since two conditions (GFR and FLC level) had to be fulfilled before stopping dialysis. Furthermore, the small number of patients, the presenting modes (relapse or de novo) and the variability of therapy preclude any conclusion relative to chemotherapy efficacy, analysis of survival and renal recovery. Only one patient presented a second episode of acute renal failure, which occurred 2 years after the first episode. Until we can monitor individual FLC nephrotoxicity thresholds, our approach seems reasonable as, in our cohort, the lowest level of initial FLC in a biopsy proven myeloma cast nephropathy was of 820 mg/L. Finally, our cohort of patients had a high early mortality that was caused by resistance to chemotherapy and chemotherapy-related complications.

In conclusion, our results suggest that in the setting of acute dialysis-dependent myeloma cast nephropathy due to elevated kappa FLC, HDF with 2.2 m^2^ heat sterilized high-flux polyphenylene HF could be used as an alternative to HCO-dialysis with a lower albumin loss. The benefits of this renal replacement modality need confirmation with a randomized controlled trial.

## Supporting Information

S1 Dataset Free light chain reduction ratio dataset. (XLSX)
